# A Rare Case of Giant Mesenteric Lipoblastoma in a 6-Year-Old Child and Review of the Literature

**DOI:** 10.1155/2020/3018065

**Published:** 2020-07-24

**Authors:** Maria Enrica Miscia, Gabriele Lisi, Giuseppe Lauriti, Angela Riccio, Dacia Di Renzo, Valentina Cascini, Pierluigi Lelli Chiesa

**Affiliations:** ^1^Department of Pediatric Surgery, “Spirito Santo” Hospital, Pescara, Italy; ^2^Department of Medicine and Aging Sciences, “G. d'Annunzio” University, Chieti-Pescara, Italy

## Abstract

Giant mesenteric lipoblastoma is a rare benign tumor arising from the adipocytes. It can mimic malignant tumors, and its diagnosis is difficult before surgery. Imaging studies could lead the diagnosis but not confirm it. Those tumors arising in the abdomen are usually larger and can cause symptoms of compression. Surgical excision is the treatment of choice, and a long-term follow-up is necessary to detect local recurrences. Only a few cases of lipoblastomas arising from the mesentery are reported in literature. We present a case of a rare giant lipoblastoma arising from the mesentery of a 6-year-old girl, with a history of postprandial abdominal pain.

## 1. Introduction

Adipose tumors are rare in childhood and represent 6% of all the neoplasms of the soft tissue.

They can be classified into malignant (liposarcoma) and benign (lipoma, lipoblastoma, lipoblastomatosis) [1, 2].

Lipoblastoma is a rare, encapsulated tumor arising from the embryonal fat tissue [3].

It is more frequent in children younger than 3 years old, with a male to female ratio of 3 : 1 [1, 3].

Common localizations are extremities and trunk. Intra-abdominal lipoblastoma is extremely rare (<7%) and mesenteric localization is exceptional [2–4].

We present a case of giant mesenteric lipoblastoma in a girl, reviewing and discussing pertinent literature.

## 2. Case Report

A 6-year-old girl came to our attention for a 2-month-lasting postprandial abdominal pain.

Her medical history was suggestive of constipation for 2 years. A painless, mildly distended, not tender abdomen was palpable on physical examination.

**Figure 1:**
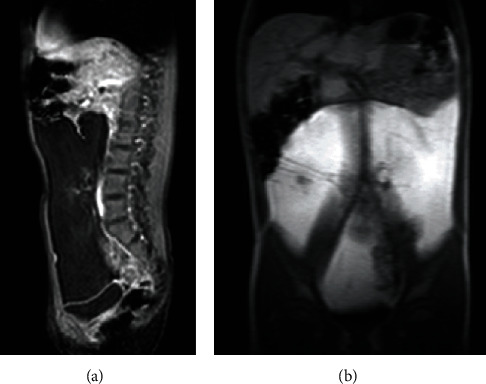
(a, b) MRI ((a) T1 weighted, (b) Thrive sequences) showing the mass occupying the entire abdominal cavity, compressing and displacing the bowel, the inferior vena cava, and common iliac veins.

An abdominal ultrasound showed a hypoechoic abdominal mass of 9 × 4 cm in size, not vascularized at the color-Doppler study and well separated from the adjacent organs. In the suspicion of a lipoma, the girl underwent an abdominal magnetic resonance imaging (MRI), which confirmed the presence of an encapsulated fatty-dense mass (20 × 4 × 18 cm), occupying the entire abdominal cavity (Figures 1(a) and 1(b)).

Blood exams (complete blood count, C-reactive protein, and liver function tests) and tumoral markers (alfa-fetoprotein, beta-human chorionic gonadotropin, lactic dehydrogenase, carcinoembryonic antigen, and neuron-specific enolase) were within ranges.

**Figure 2:**
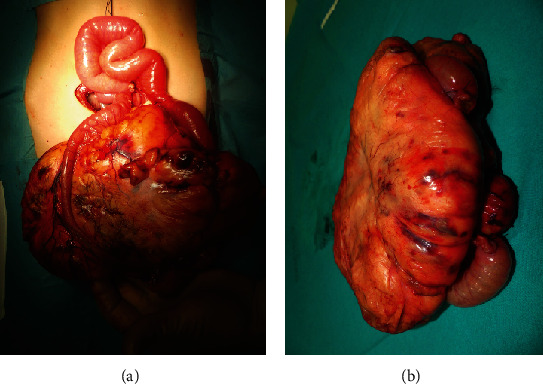
(a, b) Intraoperative findings: the mass comes from the mesentery, stretching the involved ileum (a), which was resected together with the mass (b).

An explorative laparoscopy was then performed. It showed a huge fatty mass arising from the ileal mesentery and stretching the ileal loops. The mass was completely excised through a minilaparotomy, even if a resection of a tract of intestine involved was necessary. The lesion was 21 × 19 × 7 cm and weighted 1,236 g (Figures 2(a) and 2(b)). Bowel continuity was restored through a primary end-to-end anastomosis.

Histopathological analysis showed adipocytes at different stages of maturation with focal myxoid areas and confirmed the diagnosis of giant abdominal lipoblastoma.

**Figure 3:**
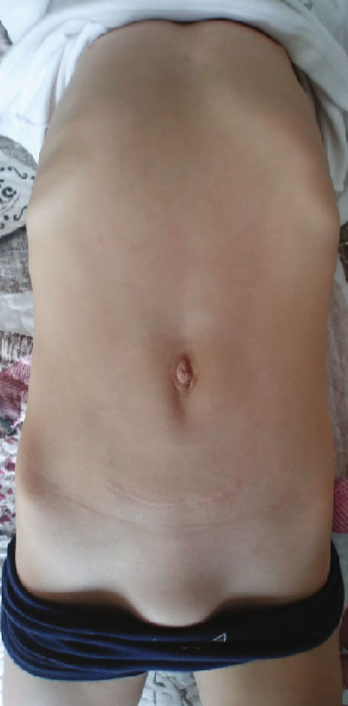
Esthetic results at follow-up.

Postoperative period was uneventful with excellent esthetic results. No recurrence was noticed at a 1-year ultrasonographic follow-up (Figure 3).

## 3. Review of the Literature

A review of the English literature was performed using a defined search strategy. Scientific databases (PubMed, Medline, Cochrane Collaboration, and Scopus) were screened looking for studies reporting on mesenteric lipoblastoma in children. MeSH headings and terms used were “lipoblastoma AND children”. Reference lists were examined to identify relevant cross-references. Of 408 titles and abstracts, 22 case reports were included, reporting data on 22 cases from 1973 to date (Table 1) [4–24]. We found a male to female ratio of 1.3 : 1 (12 M, 9 F; 1 not available). Mean age at diagnosis was 29 ± 21 months, and preferred localization, when reported, was the ileal mesentery (6/11).

Three out of 18 patients had an acute onset, with volvulus [7–9]. None of the patients underwent a laparoscopic excision of the mass. A resection of small bowel has been reported in 9/17 cases.

Mean time of follow-up was 16 ± 12 months. No recurrences have been reported, when mentioned.

## 4. Discussion

Lipoblastoma is a rare benign, encapsulated tumor of adipocytes, accounting for 5-30% of all the adipose tumors [3]. When diffuse and infiltrative, it is called lipoblastomatosis. It was firstly described by Jaffe in 1926 [4].

It arises from the abnormal proliferation of embryonic fat cells but its etiopathogenesis is not completely understood [1, 3, 25].

It is more frequent in the first decade of life, with a peak of incidence in males younger than 3 years (about 90% of all cases) [1, 3].

These tumors are usually asymptomatic; however, they can cause a mass effect when reaching considerable dimensions [3, 25].

Lipoblastoma is commonly located at the level of the trunk and extremities. Intra-abdominal localizations are rare; nonetheless, intraperitoneal tumors usually reach superior dimensions [2, 3].

Typical symptoms of abdominal lipoblastomas are abdominal pain, constipation, and vomiting, secondary to organ compression. Acute manifestations, secondary to volvulus or intussusception, are also reported, although rare [7–9].

Imaging studies, especially MRI, are useful to define the adipose nature, the extent, and tissue involvement of neoplasia. However, this study is unable to differentiate among lipoma, lipoblastoma, and liposarcoma [3, 26, 27]. In fact, the proportion of the myxoid stroma determines the imaging appearance of the lesion: a well-defined predominantly fatty lesion is likely to be a lipoblastoma, while an infiltrative lesion with a less-represented fatty component could be a lipoblastomatosis [27]. The first imaging study performed is the ultrasound; however, an MRI or a computed tomography scan is usually required to better define the adipose nature and the relationship with the surrounding organs. Definitive diagnosis is secondary to histopathological examination, and surgical excision is the treatment of choice.

Microscopic features consist of lobular architecture and myxoid areas with spindle cells and lipoblasts at various stages of differentiation [28, 29].

Rearrangements of chromosome 8q11 codifying for the oncogene PLAG1 are found in more than 70% of cases [2–4, 28–30]. The different molecular alteration found in adipocytic tumors could help in differentiating between lipoma, lipoblastoma, and liposarcoma [31]. This information could be useful to get before surgery, especially if the resection of the tumor requires a long length of intestinal resection.

Recurrence rate is reported to range between 9 and 46%, and it is usually secondary to an incomplete surgical excision [3, 6, 30].

A problem related to mesenteric lipoblastomas is the massive size of the mass and its close contiguity to the mesenteric vessels and intestinal loops, which makes a purely laparoscopic excision virtually impossible to perform. In children, laparotomy incision should be as small as possible, to limit the extent of the scar, as we did in our case.

## 5. Conclusions

Mesenteric lipoblastoma is a rare benign tumor.

It can be completely asymptomatic or can cause symptoms related to a mass effect. Imaging studies are unable to reach the definitive diagnosis; therefore, surgical excision is the treatment of choice and leads to a definitive diagnosis through the histopathological examination of the specimen.

We stress the importance of a surgery that should be as mini-invasive as possible, considering the localization and the size of the mass. A long-term ultrasonographic follow-up is necessary to detect local recurrences.

## Figures and Tables

**Table 1:** Review of the English literature.

Author	Year	Sex	Age	Symptoms	Size (cm)	Position	Surgery	Follow-up	Recurrence
Chung and Enzinger [9]	1973	F	2 yrs	n.a.	n.a.	n.a.	Excision mass+23 cm small bowel	n.a.	No
Friedman et al. [10]	1981	F	13 mo	Palpable mass	n.a.	n.a.	n.a.	n.a.	No
Stringel et al. [11]	1982	M	13 mo	Abdominal distension, palpable mass	12 × 10.8 × 17	Ileum	Excision mass+26 cm small bowel	1 yr	No
Jimenez [12]	1986	M	11 mo	Abdominal distension, palpable mass	12 × 10 × 0.8	Jejunum	Excision mass+part small bowel	3 yrs	No
Zanetti [13]	1988	F	4 yrs	Pain, vomiting	18	n.a.	Excision mass+part intestine	2 yrs	No
Denath [14]	1988	F	2 yrs	Abdominal distension	n.a.	n.a.	n.a.	n.a.	No
Prando et al. [15]	1990	M	2 yrs	Abdominal distension, palpable mass	23 × 19 × 9	n.a.	Excision mass	n.a.	n.a.
Schulman et al. [16]	1992	M	2 yrs	Abdominal distension, palpable mass	n.a.	n.a.	Excision mass	n.a.	n.a.
Posey et al. [17]	1998	M	10 mo	Abdominal distension, palpable mass	10 × 6 × 13	Ileum	Excision mass	2 yrs	No
O'Donnell et al. [18]	2000	F	5 mo	Abdominal distension, diarrhea	14.5 × 11 × 10.5	Transverse colon	Excision mass	6 mo	No
Mo et al. [19]	2003	F	16 mo	Abdominal distension, palpable mass	10 × 9 × 13	Ileum	Excision mass+26 cm small bowel	n.a.	n.a.
Al-Salem and Al-Nazer [8]	2003	M	2 yrs	Obstruction, midgut volvulus	8 × 6 × 5	Ileum	Excision mass	n.a.	n.a.
Jung et al. [20]	2005	M	17 mo	n.a.	9.7 × 7 × 5.5	n.a.	n.a.	23 mo	No
Cudnik et al. [5]	2008	M	3 yrs	Abdominal distention, fullness, intermittent pain	15 × 4.5 × 10	Jejunum	Excision mass+part small bowel	1 yr	No
Yu et al. [7]	2009	F	7 yrs	Obstruction, midgut volvulus	10 × 8 × 6	Jejunum	Excision mass+part small bowel	1 mo	No
Tang et al. [21]	2009	M	4 yrs	Intermittent abdominal pain	18 × 15 × 10	Ileocecal	Excision mass	4 mo	No
Jia and Zhang [22]	2009	M	4 yrs	n.a.	n.a.	n.a.	n.a.	n.a.	No
Gentimi et al. [4]	2011	M	18 mo	Abdominal distension, palpable mass	14 × 11 × 8	Ileum	Excision mass+part small bowel	30 mo	No
Capasso et al. [23]	2014	M	3 yrs	Abdominal pain and vomiting	n.a.	n.a.	Excision mass	n.a.	n.a.
Ghosh et al. [24]	2015	F	14 mo	Palpable abdominal mass, intestinal obstruction	n.a.	n.a.	Excision mass	n.a.	n.a.
Yang et al. [6]	2016	n.a.	n.a.	n.a.	n.a.	n.a.	n.a.	n.a.	No
Present case	2019	F	6 yrs	Postprandial abdominal pain	21 × 19 × 7	Ileum	Excision mass+part small bowel	1 yr	No

n.a.: not available.
